# Adipose Tissue Characteristics Related to Weight Z-Score in Childhood

**DOI:** 10.5812/ijem.8744

**Published:** 2013-04-01

**Authors:** Juan Jesus Haro-Mora, Eva Garcia-Escobar, Nuria Porras, Dolores Alcazar, Joaquin Gaztambide, Antonio Ruiz-Orpez, Sara Garcia-Serrano, Juan M. Gomez-Zumaquero, Eduardo Garcia-Fuentes, Juan P Lopez-Siguero, Federico Soriguer, Gemma Rojo-Martinez

**Affiliations:** 1 Carlos Haya Hospital, Endocrinology and Nutrition Department, Research Laboratory, Malaga, Spain; 2 Health Institute Carlos III, CIBERDEM, CB07/08/0019, Malaga, Spain; 3 Maternal and Child Hospital of Malaga, Pediatric Surgery Department, Malaga, Spain; 4 Health Institute Carlos III, CIBEROBN, CB06/03/0060 Malaga, Spain; 5 Maternal and Child Hospital of Malaga, Pediatric endocrinology Department, Malaga, Spain

**Keywords:** Adipose Tissue, Adipocyte, Adipokines, PPAR gamma, Adiponectin

## Abstract

**Background:**

Childhood obesity has grown very fast over recent decades and now it represents a serious public health problem. The number of adipocytes is set in childhood and adolescence and then, an effective understanding of the development of adipose tissue during these periods will help in the prevention of this pathology.

**Objectives:**

The current study aimed to determine which adipose tissue characteristics are related to a high weight Z-score in childhood.

**Patients and Methods:**

The current study included 82 children aged 5-130 months who underwent inguinal hernia surgery. Anthropometric variables were measured, and a nutritional and physical activity questionnaire was completed. Subcutaneous adipose tissue samples, taken during the operation, were analyzed for preadipocyte number, adipocyte volume, fatty acid composition (gas chromatography of FAME), and relative gene expression of various genes (real time PCR).

**Results:**

The results showed that children with a higher weight Z-score spend more time in sedentary activities and less time running or involved in active games. SCD-1 activity index, arachidonic/linoleic index, and adipocyte volume were significantly higher in children with a weight Z-score greater than 0. The preadipocyte number and the genetic expression of the studied genes did not differ between the groups. A multiple regression analysis was done to determine which variables were related to the weight Z-score. R2 values indicated that the model which included adipocyte volume, SREBP-1c, SCD-1 expression, and activity index, predicted 59% of the variability in the weight Z-score among the children. The main variables associated with adipocyte volume were PPARγ, Adiponectin, CB1R expressions, as well as the SCD-1 activity and normalized weight.

**Conclusions:**

It was concluded that in childhood, the weight Z-score is related to adipocyte volume and adipose tissue gene expression.

## 1. Background

Childhood obesity has grown very fast over recent decades and now it represents a serious public health problem. Obesity is produced by an increase in the size of fat deposits and the main factors involved in this increase are changes in lifestyle habits and in food intake, as well as a genetic predisposition (1).

Adipose tissue growth is due to two processes, hyperplasia and hypertrophy. Usually, an increase in energy intake, due to a high-energy diet or a low level of physical activity, is absorbed by adipocytes increasing their volume but, over the long term, the total storage capacity of adipocytes can be reached, and excess fat is then accumulated in other organs, like muscle, liver and heart, causing lipotoxicity and associated metabolic complications (2). An increase in adipocyte number, or hyperplasia, is due to the differentiation of preadipocytes into adipocytes (3), and it has been hypothesized that since there is a higher potential number of adipocytes that can store more fat, a greater number of preadipocytes can prevent lipotoxicity (4). The number of adipocytes is set in childhood and adolescence, remaining constant in adulthood (5). Overweight or obese children are more likely to develop obesity in adolescence and adulthood (6, 7). Therefore, effective prevention and treatment can be achieved by a better understanding of the pathogenesis of obesity, in particular determining whether physical activity or lifestyle have an effect on adipose tissue gene expression in the early stages of the development of this tissue.

Certain genes related to adipose tissue development produce an increase in hyperplasia, raising the number of adipocytes, and thus allowing a greater but safe accumulation of fat (4). Genes that contribute to preventing lipotoxicity include PPARγ and SREBP-1c. These genes are transcription factors influencing the expression of genes which promote the adipogenic pathways. PPARγ serves as the master transcriptional regulator of adipogenesis (8) and it is therefore related to adipose tissue development. SREBP-1c mediates the transcriptional effects of insulin on genes, encoding enzymes involved in glycolysis, lipogenesis and gluconeogenesis (9).Other proteins also have an important role in lipid metabolism. Stearoyl Co-A desaturase (SCD-1), is involved in the desaturation of fatty acids, and its activity can alter the composition of phospholipids and triglycerides. Its effect on phospholipid composition can alter the fluidity of the plasma membrane, and these alterations have been implicated in a variety of disease states (10). These three proteins can be affected by dietary PUFAs (11), and there is a link between their functions and food intake. Other genes related with the adipose tissue are adiponectin, an insulin-sensitizing protein that also promotes fatty acid oxidation (12), insulin receptor and insulin receptor substrate 1 (IRS-1), key proteins from the insulin signalling pathway (13), and cannabinoid receptor 1 (CB1R) and osteocalcin, which are related with energy metabolism (14, 15).

## 2. Objectives

Few studies have examined the relationships between adipose tissue gene expression, environmental factors and adiposity in children. Consequently, the current research aimed to study characteristics of adipose tissue, including the expression of certain genes, in relation to a high weight Z-score in childhood.

## 3. Patients and Methods

The study included 82 consecutively selected children aged 5-130 months (22 girls and 60 boys), who underwent inguinal hernia surgery. Children with any other disorder or in pubertal status were excluded from the study. The sample size was calculate assuming an alpha equal to 0.05, beta equal to 0.9 and an error of 20%, and the minimum number of subjects to study the adiposity and the relation to gene expression was 52 subjects. After obtaining consent from the parents, a sample of subcutaneous fat, weighing 40-120 mg, was taken during the operation from the area of the inguinal incision. The sample was studied fresh.

All the children underwent an anthropometric study and questionnaires were completed by their parents about physical activity and food intake.

### 3.1. Procedures

The weight was standardized for age and sex (Z-scores) based on published weight tables for Spanish children (16). Data were recorded on the time spent sleeping, resting, lying down, sitting, walking and running, and an activity index was calculated (time spent walking and running/24h). The nutritional study was done using a questionnaire that collected information on the frequency of consumption of the main foods per week. The adipose tissue fatty acid composition, the adipocyte volume and the number of preadipocytes were determined as described in a previous publication by the authors (17).

Total RNA was extracted from subcutaneous adipose tissue using Trizol. First strand cDNA was synthesized by retrotranscription using the M-MLV retrotranscriptase (Promega). Gene expression levels were analyzed by quantitative real-time reverse transcriptase polymerase chain reaction (RT-PCR) using an Opticon II Real Time PCR system (MJ Research). The primers used in the experiments were designed with the online program Primer3. Calculation of relative expression levels of the different transcripts was performed based on the cycle threshold (CT) method. Standard curves were constructed for the genes studied and β-actin (internal control). The CT value for each sample was determined using Miner (18) and then it was used to calculate the amount of SREBP-1c, PPARγ, SCD-1, adiponectin, insulin receptor, IRS1, CB1R and osteocalcin. The expression of each gene was relativized to the expression of βactin, using the ΔΔCt method (19).

### 3.2. Statistical Study

The data were expressed as the mean ± SD, median interquartile ranges, or proportions. Comparisons of means were made by t-test with parametric variables, and by Mann Whitney test with non parametric variables. The association between variables was evaluated by multiple linear regression models. Non-parametric variables included in the regression models, were transformed using logarithms.

## 4. Results

Table 1 shows various characteristics of the children according to their weight, whether it was below or above the age- and sex-standardized mean weight (Z-score). Both groups had the same age and sex ratio, but the children with a higher weight Z-score spent more time in sedentary activities and less time running or in active games, with the result that the activity index was 22.2% lower than in the children with a weight Z-score below 0.

**Table 1.** Mean Variables According to the Weight Z-Score in the Two Groups.

	Weight Z score ≤0, N=52	Weight Z score >0, N=30	P Value
**Age, mo **	46.9 ± 35.7	50.2 ± 36.8	0.5
**Sex, male, %**	75.7	78.9	0.5
**Activity index, %**	26.6 ± 15.1	20.7 ± 12.6	0.005
**SCD activity (palmitoleic [Table-fn fn1767]/palmitic)**	0.14 ± 0.04	0.16 ± 0.04	0.007
**Arachidonic/linoleic index**	0.011 ± 0.008	0.015 ± 0.009	0.02
**Adipocyte volume, pL ^ a^**	200.1 ± 70.8	324.7 ± 102.6	< 0.001
**Nº preadipocytes per gram of tissue x 10^5^** **^a^**	32.5 ± 26.9	29.5 ± 24.3	0.8
**Adiponectin expression ** **^ a^**	0.56 ± 0.15	0.54 ± 0.15	0.2
**CB1R ** **^ a^**	0.55 ± 0.26	0.61 ± 0.29	0.9
**Insulin receptor ** **^ a^**	0.51 ± 0.21	0.52 ± 0.18	0.4
**IRS1 ** **^ a^**	0.48 ± 0.26	0.57 ± 0.21	0.7
**PPARγ** **^ a^**	0.84 ± 0.27	0.66 ± 0.23	0.2
**SCD1 ** **^ a^**	0.46 ± 0.30	0.50 ± 0.23	0.5
**SREBP-1c ** **^ a^**	0.54 ± 0.33	0.96 ± 0.43	0.7
**Osteocalcin** **^ a^**	0.66 ± 0.35	0.70 ± 0.38	0.5

^a^Data are expressed as median and semi-interquartile ranges, p = significance level of Mann-Whitney test.

Abbreviations: CB1R, cannabinoid 1 receptor; IRS1, insulin receptor substrate 1; PPAR, peroxisome proliferator activated receptor gamma; SCD1,stearoyl-CoA desaturase 1; SREBP-1c,stearoyl responsive element binding protein

The SCD activity index, arachidonic / linoleic index, and adipocyte volume were significantly higher in the children with a weight Z-score greater than 0, reflecting a different adipose tissue fatty acid composition. The preadipocyte number and the genetic expression of the studied genes were similar in the two weight Z-score groups. The number of preadipocytes correlated inversely with the adipocyte volume (R^2^ = -0,19; P = 0.015). To determine which variables were related with the weight Z-score authors performed a multiple regression analysis. R^2^ values indicated that the model predicted 59% of the variability in the weight Z-score among the children (Table 2, model 1). The expression of SCD-1 and SREBP-1c was directly related in non-adjusted models (r = 0.37; P < 0.0001), and after adjusting for age (r = 0.19; P = 0.04), although in the complete adjusted model the relationship between their expression and the weight was inverse.

**Table 2.** Linear Regression Models

Model 1.^ a^ Depedent variable: weight^ b^ Z-score, R^2^=0.59	β-Standarized	P Value
**Adipocyte volume ** ^ c^	0.50	< 0.001
**SREBP-1c expression** ^ c, d^	0.57	< 0.001
**Activity index**	0.29	0.006
**SCD-1 ** **expression** ^ c^^,^^ d^	-0.30	0.02
**Model 2. a Dependent variable: adipocyte volume ^c^ R^2^=0.59**		
**Normalized weight** ^ d^	0.43	< 0.001
**SCD-1 activity**	0.27	0.009
**Adiponectin expression** ^c, d^	-0.43	< 0.001
**CB1 receptor expression** ^ c, d^	0.37	0.001
**PPARg expression** ^ c, d^	-0.33	0.003

^a^Variables related to the weight Z-score (model 1) and to adipocyte volume (model 2). Method: stepwise. Variables included in each analysis: Model 1: adipocyte volume, preadipocyte number, SREBP-1c expression, PPARg expression, CB1 receptor expression, Adiponectin expression, SCD-1 expression, INS receptor expression, IRS1 expression, osteocalcin expression, activity index. Model 2: weight Z-score, preadipocyte number, SREBP-1c expression, PPARg expression, CB1 receptor expression, Adiponectin expression, SCD-1 expression, INS receptor expression, IRS1 expression, osteocalcin expression, activity index.

^b^Weight normalized for gender and age

^c^These variables were transformed using logarithms

^d^The gene expressions are relativized to the expression of β-actin

The expression of PPARγ, Adiponectin, CB1R and, as well as the SCD-1 activity and normalized weight, with the latter five factors explaining 59% of the variability found in the adipocyte volume (Table 2, model 2).

Food did not modify the relationship observed (data not shown).

## 5. Discussion

The main finding was that the adipose tissue expression of a few genes explained more than half of the variability among the children in the weight. Children with a weight above the mean for their age and sex were less active, had a larger adipocyte size, and had a different adipose tissue fatty acid composition. However, the gene expression profile did not differ between the groups, at least for the genes studied here.

Nevertheless, when considering the effect of all the genes simultaneously together with the adipocyte volume and the number of preadipocytes, up to 68% of the weight variance was explained by a model containing the relative expression of two genes, SREBP-1c and SCD-1. The relative expression of SCD-1 is inversely related to children’s weight while the SCD-1 activity is directly related to the size of the adipose cell. The relation between SREBP-1c and SCD-1 in adipose tissue is not completely known. It has been hypothesized that the expression of SCD-1 in this tissue could be regulated by other pathways, independently of SREBP-1c, like ChREBP or C/EBPα (20).

Regarding the cellularity of the subcutaneous adipose tissue, in the current study the adipocyte volume was inversely associated with the expression of adiponectin, a known insulin sensitizing hormone (21), and with the expression of PPARγ, which is the master of the adipogenesis pathway (12). PPARγ stimulates the formation of new adipose cells, therefore encouraging hyperplasia instead hypertrophy. Nonetheless, there was no significant relation between the number of preadipocytes and the relative expression of the studied genes. Other studies have demonstrated that the expressions of PPARγ and SREBP-1c are positively associated with BMI in obese women (22), indicating a growth of adipose tissue due to hyperplasia. In the present study, both adipocyte volume and SREBP-1c expression were positively related to weight, which may indicate that hyperplasia or hypertrophy could be contributing to the growth of adipose tissue in children. There was no relation between weight and PPARγ expression in adipose tissue, though PPARγ expression was negatively associated with adipocyte size, as well as adiponectin.

In obese adult humans, subcutaneous abdominal adipose cell enlargement is characterized by a reduced number of preadipocytes which can undergo differentiation (23). In the current study, adipocyte volume was inversely related to preadipocyte number. In periods of a positive energy balance, the high number of preadipocytes may facilitate the creation of new adipose cells, increasing the size of adipose cells unnecessarily, and therefore limiting the hypertrophic growth of adipose tissue (4). This could explain the negative relation found between the number of preadipocytes and adipocyte volume. Small adipocytes are more insulin-sensitive and have a high avidity for the uptake of free fatty acids and triglycerides, preventing their deposition in non-adipose tissue (24). However, Liu et al found that a small adipocyte cell size is associated with insulin resistance in morbidly obese women (25). The reason for this apparent contradiction may be that the small adipocyte size acts as a compensatory mechanism of the insulin resistance in these patients.

The expression of the CB1 receptor was directly related to the adipocyte size in the current research data. In both animal and human obese models there was an over expression of the CB1 receptor in several organs, including adipose tissue (26), however some studies have shown a lower expression in lean subjects (27, 28). CB1receptor expression changes according to the level of differentiation of the adipocytes, it is low in the state of preadipocytes and high when the adipocytes reach maturation (26). However, no data is given in these publications about preadipocyte number, which could be considered a determinant of the CB1 receptor expression in subcutaneous adipose tissue, as possibly indicated by the current study results.

The current study detected the expression of osteocalcin in the subcutaneous adipose tissue of children for the first time. The expression of osteocalcin in adipose tissue has been described in adult obese males (15), but never in children, whether obese or not. Some authors consider osteocalcin similar to a hormone, able to increase adiponectin expression, β–cell proliferation and insulin secretion, as well as energy expenditure (29). Others have found a positive relation between the levels of circulating osteocalcin and adiponectin in serum, and insulin sensitivity (30). Further studies are necessary to discern possible relations between the expression of osteocalcin in adipose tissue and other metabolic variables.

The current study has several strengths and limitations. The strong points include the simultaneous measurement of adipocyte volume and the number of preadipocytes. On the other hand, although some of the confounding variables, like physical activity or the intake of other foods were controlled, there may be other confounding variables such as the cultural level of the parents, which were not assessed in this study. No quantitative nutritional data are available. Moreover only subcutaneous adipose tissue was available, so we cannot rule out that weight Z-scores are also related to the properties of visceral adipose tissue. Although visceral adipose tissue is metabolically more active and is more related to metabolic diseases in adulthood (24), a high intake of food causes hypertrophy of the subcutaneous tissue, which may be the source of metabolic impairment of other tissues, including visceral adipose tissue (31). Another limitation concerns the small size of the biopsy samples obtained, which prevented the measurement of proteins or more genes.

It can be concluded that, in childhood, the weight Z-score is related to adipocyte volume and adipose tissue gene expression.
